# Congenital Vomer Agenesis: Report of Two Cases 

**Published:** 2017-05

**Authors:** Mehdi Bakhshaee, Sherwin Tavakol, Yeganeh Teimouri

**Affiliations:** 1 *Sinus and Surgical Endoscopic Research Center, Faculty of Medicine, Mashhad University of Medical Sciences, Mashhad, Iran.*; 2 *Department of Otorhinolaryngology, Mashhad University of Medical Sciences, Mashhad, Iran.*

**Keywords:** Congenital, Endoscopic examination, Nasal septum, Vomer agenesis

## Abstract

**Introduction:**

Congenital vomer agenesis is an extremely rare condition in which the vomer bone does not fully develop, which can lead to septal perforation.

**Case Report:**

We report two cases with a defect in the vomer bone in the posteroinferior portion of the septum, found accidentally while performing a pre-operative CT scan for nasal obstruction evaluation. They were diagnosed with congenital vomer agenesis.

**Conclusion:**

There are afew reports of vomer agenesis in literatures. By increasing usage of sinonasal endoscopic examination,we expect to address more cases in the future.

## Introduction

The nasal septum is made up of crests of both the palatine and the maxillary bones, the perpendicular plate of the ethmoid bone, the quadrangular septal cartilage, as well as the vomerand its two point ossification centers (with one on either side)(1). The palatal shelves shift at either side of the inferior edge of the precartilaginous nasal septum with the nasopalatine nerve on the 8th gestational week(2).A U-shaped bony formation is formed due to the fusion of the paired bony centers on both sides of the septal cartilage on the 17^th^ week of gestation. During the 19^th^ through the 23^rd^ gestational weeks, the U-shape transforms to a Y-shaped vomeral bone (3). In subsequent fetal stages of development, the radial spread of bony trabeculae leads to lateral widening of the inferior parts of the vomer. During development, the vomer’s mineralization foci begin to appear subsequent to the fusing of the cartilaginous nasal septum with the palatal shelves (2). The ossification of the vomer begins prior to that of the perpendicular plate, directly after coming into contact with the ossification line next to the vertical plane to a horizontal plane to meet the nasal septum just above the tongue (4). Agenesis of the vomer bone is a rare condition that can lead to a defect in the posterior of nasal septum, but more common causes include trauma, irritation, tuberculosis, infection, irritation, neoplasia, and chronic inflammatory diseases (5).In vomer agenesis the defect is normally located in the posteroinferior region of the septum. In this report we want to describe a rare condition that surgeon may incidentally encountered and should be differentiate from septal perforation and defect.

## Cases Report

**Fig1:**
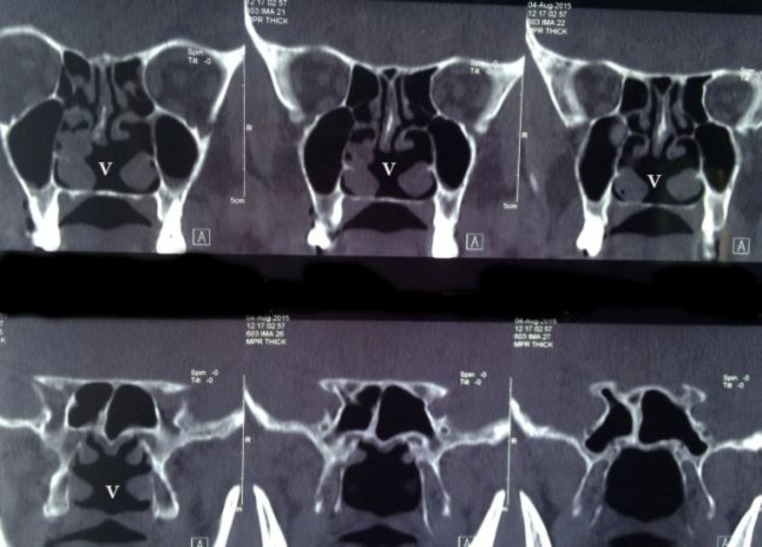
CT Scan of case 1 that shows a defect in posteroinferior part of septum. This part is in accordance to vomerian part of nasal septum (V

Case 1: Patient was 48-year-old man who presented with a 10-year history of bilateral nasal obstruction. He did not have any history of trauma, nasal surgery, cauterization, tuberculosis, or syphilis. A clinical endoscopic examination detected bilateral mild polyposis and anterior septal spur in the left side. Further examination of the ear, oral cavity, and pharynx were normal. In a pre-operative CT scan we found a defect in the posteroinferior portion of the septum and bilateral sinonasal polyposis (Fig.1). 

**Fig 2:**
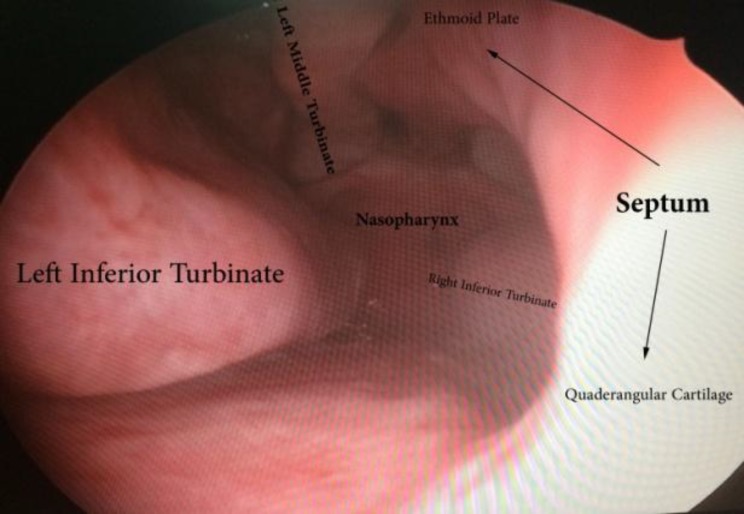
Endoscopic view of septal nasal defect with neighbor structure and smooth mucosal margin

After removing the polyps during surgery, we were able to identify the nature of this defect which compatible with vomerian part of nasal septum. The margins were smooth and the mucosa was intact and normal (Fig.2). 

This condition was compatible with vomer agenesis.After surgery we examined the posterior part of soft palate from the point of the occult palate and there was an ovular muscle that we could ruled out submucosalceleft palate.

**Fig3:**
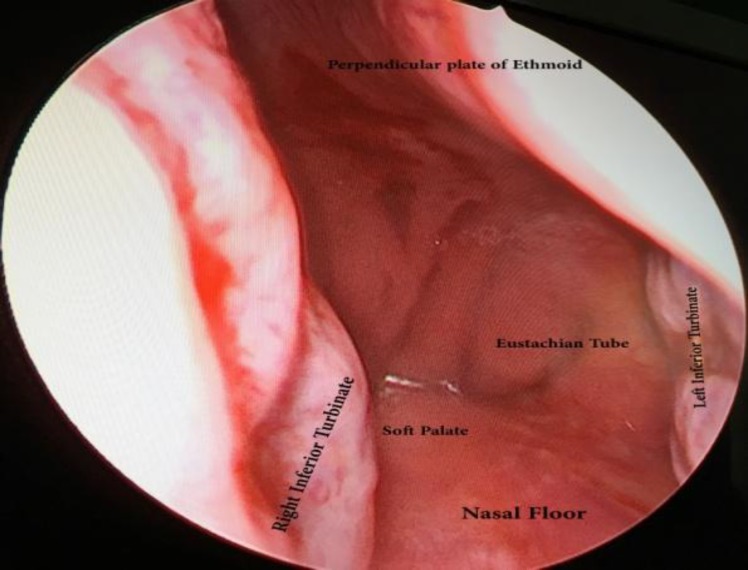
Endoscopic view of smooth posteroinferior nasal septum defect with intact margin and other nasal structures.

Case 2: Another case was a 27-year-old man with congenital maxillofacial deformity who was a candidate for nasal reconstruction from the points of cosmetic as well as functional view. He did not have any history of trauma, surgery or systemic diseases. During endoscopic examination, we found a bilateral narrowing of the nasal valve area and a defect in the posteroinferior part of the septum with smooth margins and healthy mucosa around it (Fig.3). 

**Fig 4:**
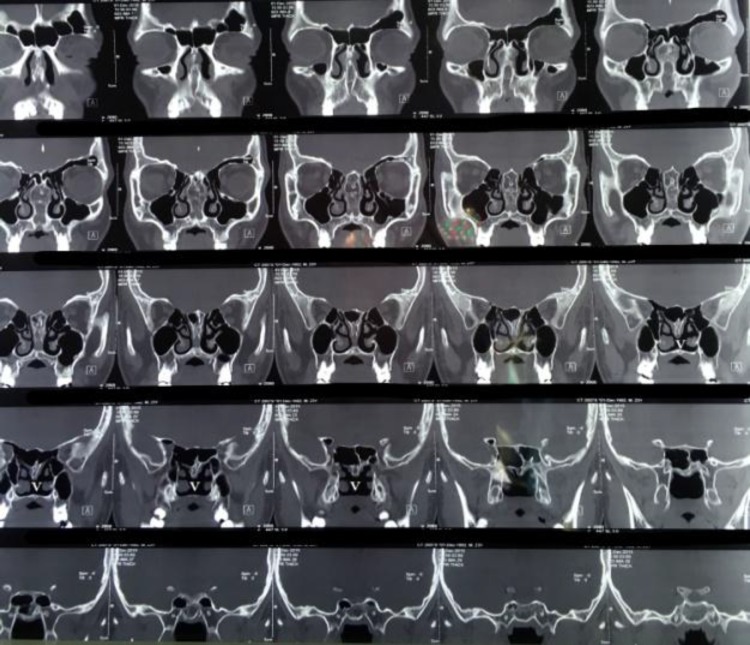
CT scan of case 2 that shows posteroinferior nasal septum defect in proposed vomerian part (V) and also thickening of the rest of septum

There was not any submucosalceleft palate. A preoperative CT scan showed and confirmed this defect in the postereoinferior part of the septum and septal thickening (Fig.4).

## Discussion

The vomer and the perpendicular plate of the ethmoid form the osseous septum. The vomer is created by intramembranous ossification, while the ethmoid bone forms via endochondrial ossification (6). During development, the vomer’s mineralization foci appear subsequent to the fusing of the cartilaginous nasal septum with the palatal shelves (2). The ossification of the vomer begins prior to that of the perpendicular plate, directly after coming into contact with the ossification line next to the vertical plane to a horizontal plane to meet the nasal septum just above the tongue (4). Mineralization of the vomer will be interrupted by any suspending of the ossification line to meet with the mesenchymal tissue of the future vomer (3). This theory is known as the “Incomplete Touch Theory,” whichdescribes the incomplete joining of the ossification line of the septal cartilage with the nearby tissue (7). Another theory proposed by Mohri is immature ossification and incomplete downward of the vomer itself (8).

Vomer agenesis has been described in the literature frequently with other concomitant diseases. Many of these are otolaryngological, but the largest concomitant disease type in patients with vomer agenesis in the literature has been ontological. Of the 6 patients that Mohri and Amatsu reported of in 2000, three of them had otitis media, and another had cholesteatoma (8). 

Yilmaz and Altuntas also report of a 19-year-old man with bilateral otitis media with effusion, as well as hypertrophy of the posterior ends of both turbinates, septal deviation, and adenoid hypertrophy (9). Other reports commonly show vomer agenesis developing with concomitant sinonasal diseases. For example, both Lee and Kang et al. have reported of patients with concomitant sinusitis (10,11). Yorgancila et al. reported a 28-year-old woman with vomer agenesis who had a maxillary retention cyst (12). In 2015, Ucar et al. reported two cases of vomer agenesis with a concomitant deviated septum and inferior turbinate hypertrophy (13). The reports of concomitant sinonasal conditions coincide more closely with the case report outlined in this paper. The literature only shows one case of a concomitant laryngeal condition (laryngeal polyp), which was reported by Mohri and Amatsu in 2000 (8).There are also a group of patients who had vomer agenesis with concomitant non-otolaryngological diseases. For example, Mohri and Amatsu reported in 2000 of a 39-year-old man with pituitary adenoma (8). Additionally, in 2004, Dogrue et al. reported 2 cases of vomer agenesis in the south of Turkey where there is a high prevalence of thalassemia. One had thalassemia and another had thalassemia accompanied with sensorineural hearingloss (14).

 Finally, there have been multiple articles that describe patients without any concomitant disease (12,13,15). Additionally, in 2012, Verim et al. proposed a hereditary basis for isolated congenital vomer agenesis. They discovered the same rare condition in the father, uncle, and two siblings of the same patient. Verim et al. had 5 cases at first and 9 cases after discovering the aforementioned ones. They proposed that this anomaly could possibly be attributed to a multifactorial hereditary disease (16). All reported cases is listed on Table 1.

**Table 1:** A list of reported cases by different authors

**Author**	**Sex**	**Age**	**Concomitant disease**
Mohn and Amatsu, 2000	F	44	Chronic otitis media
	M	55	Laryngeal polyp
	M	61	Acute otitis media
	M	4	Otitis media with effusion
	M	39	Pituitary adenoma
	F	24	Cholesteatoma
Dogru et al, 2004	M	16	Thalassemia
	F	43	Thalassemia and sensorineural hearing loss
Yilmaz and Altunta, 2005	M	19	Otitis media with effusion
Lee, 2006	M	10	None
	F	62	Maxillary sinusitis
Kang et al, 2007	M	13	Chronic sinusitis and nasal polyp
HerreroCalvo et al, 2008	F	43	None
Yorgancllar et al, 2012	M	28	Retention cyst in the maxillary sinus
Ozlay et al , 2013	F	14	Nasal Obstruction
Ucar et al , 2015	M	34	Septal deviation ,inferior turbinate hypertrophy
	M	34	Maxillofacial Truma
Bakhshaee et al, 2016	M	48	Sinonasal polyposis &Septal deviation
	M	27	Congenital midline maxillofacial deformity

## Conclusion

We know that this defect is likely more prevalent than that which has been reported in the literature, and by increasing the usage of sinonasal endoscopic examinations in otolaryngology we expect to find more cases in the future. The prevalence of concomitant otolaryngological diseases in these patients could suggest a related mechanism between the development of such diseases and vomeragenesis.
